# Neuropathological diagnoses and clinical correlates in older adults in Brazil: A cross-sectional study

**DOI:** 10.1371/journal.pmed.1002267

**Published:** 2017-03-28

**Authors:** Claudia K. Suemoto, Renata E. L. Ferretti-Rebustini, Roberta D. Rodriguez, Renata E. P. Leite, Luciana Soterio, Sonia M. D. Brucki, Raphael R. Spera, Tarcila M. Cippiciani, Jose M. Farfel, Alexandre Chiavegatto Filho, Michel Satya Naslavsky, Mayana Zatz, Carlos A. Pasqualucci, Wilson Jacob-Filho, Ricardo Nitrini, Lea T. Grinberg

**Affiliations:** 1 Brazilian Aging Brain Study Group, University of São Paulo Medical School, São Paulo, Brazil; 2 Division of Geriatrics, University of São Paulo Medical School, São Paulo, Brazil; 3 Department of Medical Surgical Nursing, University of São Paulo Nursing School, São Paulo, Brazil; 4 Department of Pathology, University of São Paulo Medical School, São Paulo, Brazil; 5 Department of Neurology, University of São Paulo Medical School, São Paulo, Brazil; 6 Department of Epidemiology, School of Public Health, University of São Paulo, São Paulo, Brazil; 7 Human Genome and Stem Cell Center, Biosciences Institute, University of São Paulo, São Paulo, Brazil; 8 Memory and Aging Center, University of California San Francisco, San Francisco, California, United States of America; University of Cambridge, UNITED KINGDOM

## Abstract

**Background:**

Clinicopathological studies are important in determining the brain lesions underlying dementia. Although almost 60% of individuals with dementia live in developing countries, few clinicopathological studies focus on these individuals. We investigated the frequency of neurodegenerative and vascular-related neuropathological lesions in 1,092 Brazilian admixed older adults, their correlation with cognitive and neuropsychiatric symptoms, and the accuracy of dementia subtype diagnosis.

**Methods and findings:**

In this cross-sectional study, we describe clinical and neuropathological variables related to cognitive impairment in 1,092 participants (mean age = 74 y, 49% male, 69% white, and mean education = 4 y). Cognitive function was investigated using the Clinical Dementia Rating (CDR) and the Informant Questionnaire on Cognitive Decline in the Elderly (IQCODE); neuropsychiatric symptoms were evaluated using the Neuropsychiatric Inventory (NPI). Associations between neuropathological lesions and cognitive impairment were investigated using ordinal logistic regression. We developed a neuropathological comorbidity (NPC) score and compared it to CDR, IQCODE, and NPI scores. We also described and compared the frequency of neuropathological diagnosis to clinical diagnosis of dementia subtype. Forty-four percent of the sample met criteria for neuropathological diagnosis. Among these participants, 50% had neuropathological diagnoses of Alzheimer disease (AD), and 35% of vascular dementia (VaD). Neurofibrillary tangles (NFTs), hippocampal sclerosis, lacunar infarcts, hyaline atherosclerosis, siderocalcinosis, and Lewy body disease were independently associated with cognitive impairment. Higher NPC scores were associated with worse scores in the CDR sum of boxes (β = 1.33, 95% CI 1.20–1.46), IQCODE (β = 0.14, 95% CI 0.13–0.16), and NPI (β = 1.74, 95% CI = 1.33–2.16). Compared to neuropathological diagnoses, clinical diagnosis had high sensitivity to AD and high specificity to dementia with Lewy body/Parkinson dementia. The major limitation of our study is the lack of clinical follow-up of participants during life.

**Conclusions:**

NFT deposition, vascular lesions, and high NPC scorewere associated with cognitive impairment in a unique Brazilian sample with low education. Our results confirm the high prevalence of neuropathological diagnosis in older adults and the mismatch between clinical and neuropathological diagnoses.

## Abstract 2

In a postmortem analysis, Lea Grinberg and colleagues examine neuropathological diagnoses in a sample of older adults from Brazil.

## Author summary

**Why was this study done?:**

- Fifty-eight percent of people with dementia live in low- and middle-income countries (LMICs), and this percentage is expected to increase to 71% by 2050.
- Definitive diagnosis of dementia etiology requires a neuropathological exam.
- In high-income countries (HICs), Alzheimer disease (AD) is the most common cause of dementia, usually coexisting with other neurodegenerative and vascular lesions.
- Almost no neuropathological information is available for LMICs; therefore, we investigated the frequency of neurodegenerative and cerebrovascular lesions and their correlation with cognitive and neuropsychiatric symptoms in a clinicopathological study with 1,092 admixed older participants from Brazil.

**What did the researchers do and find?:**

- In a cross-sectional study, we described the frequency of the most common causes of dementia, as well as the correspondence between clinical and neuropathological diagnosis, in a large population-based series with 1,092 older adults from a LMIC.
- We developed a comorbidity neuropathological score to account for the association between the coexistence of cerebral lesions and cognitive and neuropsychiatric symptoms.
- Forty-four percent of participants had enough neuropathological lesions to fulfill the criteria for at least one neuropathological diagnosis. AD was the most common diagnosis (50%), followed by vascular dementia (35%).
- Higher neuropathological comorbidity associated with worse cognition and a higher burden of neuropsychiatric symptoms.

**What do these findings mean?:**

- In a large sample from a LMIC, dementia subtypes were similar to those seen in series from HICs.
- Although vascular dementia was not as frequent as AD, its frequency was relatively higher than in HIC series.

## Introduction

In 2010, 35.6 million people lived with dementia worldwide. This number is expected to double every 20 y and reach 115.4 million in 2050 [1]. Much of this increase will be due to a surge in dementia prevalence in low- and middle-income countries (LMICs). By 2050, 71% of people with dementia will live in LMICs, as oppose to 58% in 2010 [1]. Alzheimer disease (AD) is the leading cause of dementia worldwide, according to epidemiological studies, accounting for an average of 60% of cases in LMICs [2] and ranging from 50% to 84% of cases in Latin America [3].

The neuropathological changes underlying dementia are broad and comprise neurodegenerative and non-neurodegenerative conditions. Neurodegenerative conditions are classified by their molecular signature, represented by deposits of unfolded proteins, and the distribution of these proteins. For instance, AD features deposits of beta-amyloid extracellular plaques and intraneuronal fibrillary tangles enriched for phospho-tau species [4]. Lewy body dementia shows intraneuronal deposits of alpha-synuclein, known as Lewy bodies. Frontotemporal lobar degeneration (FTLD) encompasses a large number of distinct neuropathological entities that share superficial cortical vacuolation, gliosis, and neuronal loss [5]. FTLD may feature deposits of transactivation response DNA-binding protein of 43 kDa (TDP-43) in a variety of distribution called types A to D [6]. FTLD with TDP-43 inclusions (FTLD-TDP) may overlap with motor neuron disease, especially FTLD-TDP type B. Deposits of phospho-tau protein comprise the second main FTLD type. These tau deposits are in some cases enriched for 4-repeat tau or 3-repeat tau species. Among the FTLD-tau, the most common are corticobasal degeneration, progressive supranuclear palsy, and Pick disease. Among the non-neurodegenerative causes of dementia in senior adults, cognitive changes due to a variety of different cerebrovascular lesions are the most prevalent [7,8].

Clinicopathological studies, the gold-standard for determining the underlying cause of a dementing illness, point to the fact that vascular dementia (VaD), either alone or associated with AD, is at least as common as ‘‘pure” AD in high-income countries (HICs) [9,10]. VaD is greatly associated with cardiovascular risk factors (e.g., hypertension, diabetes mellitus, and dyslipidemia). Since such factors are less likely to be detected and treated in LMICs [11], it is reasonable to suppose that cerebrovascular disease causes more injuries in these countries than in HICs. In the first community-based clinicopathological study on dementia in a population from a LMIC (São Paulo, Brazil) [12], we found that VaD in isolation or overlapping with AD underlaid over one-third of the 113 dementia cases. Unfortunately, that sample size was too small for exploring the influence of other factors distinguishing HICs from LMICs that impact the clinical expression of dementia, such as low educational attainment [13] and different race composition [14–16]. For the same reason, the small sample size failed to offer enough power for examining the role of the coexistence of different neuropathological lesions contributing to cognitive impairment [9,10,17–21].

Here, we investigated the underlying causes of dementia, clinicopathological correspondence, and the association of specific neuropathological lesions in groups and in isolation with cognitive and neuropsychiatric symptoms in 1,092 participants of admixed races, of the Brain Bank of the Brazilian Aging Brain Study Group (BBBABSG) [22].

## Methods

### Participants

Autopsy verification is mandatory in Brazil to define the cause of death for most individuals who die of natural causes. The São Paulo Autopsy Service (SPAS) is the only morgue serving the metropolitan area of São Paulo (Brazil). Eligible participants to the BBBABSG have the brain donated by the deceased’s next-of-kin after death upon the signature of informed consent. All BBBABSG protocols, the informed consent form, and procedures follow international and Brazilian regulations for research involving humans and were approved by the local and federal research committees. A detailed description of the BBBABSG procedures can be found elsewhere [22].

This cross-sectional study includes participants recruited from 2004 to 2014. Enrollment eligibility includes age of death above 50 y and the presence of a knowledgeable informant to provide clinical and functional information. A knowledgeable informant was someone who had at least weekly contact with the deceased in the last 6 mo before death. Individuals were excluded if clinical data were inconsistent or if the brain tissue was incompatible for neuropathological analyses (e.g., cerebrospinal fluid pH < 6.5 or major acute brain lesions including hemorrhages) [22]. This study is reported as per Strengthening the Reporting of Observational Studies in Epidemiology (STROBE) guidelines (S1 Checklist).

### Clinicofunctional assessments and definitions

Trained gerontologists supervised by a registered nurse with expertise in dementia performed the clinicofunctional assessments. After the informed consent had been signed, the most knowledgeable informant was interviewed to obtain the deceased’s past clinical history using a semistructured interview, which was previously validated for postmortem use and has shown good evidence of validity for the detection of cognitive impairment by informants in postmortem settings [23]. The clinicofunctional assessment lasts for about 40 min and consists of three parts:

1. Sociodemographics (age at death, sex, years of formal education, race, and the frequency of contact with the informant). Race was reported by the next-of-kin during the clinical interview, according to the following categories: white, black, brown, and other races (i.e., Asian and Brazilian Indian). Other demographic data were confirmed on government-issued documents;
2. Past medical history (hypertension, diabetes, coronary artery disease, heart failure, arrhythmia, and stroke), family history, and lifestyle data (smoking habits, alcohol use, and physical activity);
3. Clinicofunctional and neuropsychiatric assessment. Cognitive impairment was assessed using the Clinical Dementia Rating (CDR) [24] scale and the Informant Questionnaire on Cognitive Decline in the Elderly (IQCODE) [25]. The CDR is a five-point scale used to stage dementia severity by assessing six domains: memory, orientation, judgment and problem solving, community affairs, home and hobbies, and personal care. Because of the nature of this study, only the informant section of the CDR was applied. Participants were then classified into five categories: normal cognition (CDR 0); questionable dementia (CDR 0.5); mild dementia (CDR 1); moderate dementia (CDR 2); and severe dementia (CDR 3). Given that the CDR is a categorical scale, we also used the continuous CDR sum of boxes (CDR-SOB) scores ranging from 0 to 18 points in part of our analysis [26]. The retrospective form of the IQCODE was also applied as an alternative measure of cognitive function. The IQCODE’s 26 items assess whether the person had changes in cognition compared to 10 y ago and result in scores from 1 to 5. A score of 3 means no change, and a 5 means that an individual declined considerably in all items evaluated. The IQCODE has been widely validated for the Brazilian population [23,27]. Neuropsychiatric symptoms were assessed using the Neuropsychiatric Inventory (NPI) [28], which evaluates frequency and severity of a wide range of neuropsychiatric symptoms (delusions, hallucinations, dysphoria, anxiety, agitation, aggression, euphoria, disinhibition, irritability, lability, apathy, and aberrant motor activity). Scores vary from 0 to 144 points, and higher scores are associated with higher frequency and severity of symptoms. Upon autopsy, we also measured the deceased’s weight and height without clothes in the supine position, using an electronic scale and a stadiometer, respectively. Body mass index was calculated by dividing the weight in kilos by the square of the height in meters. In participants with CDR ≥ 1, the clinical diagnosis of the dementia subtype was defined by a panel consensus including a geriatric nurse with expertise in dementia (RELFR), the nurse supervisor (LS), and two neurologists (RN and SMDB). Dementia diagnosis followed the definitions from the fourth edition of the Diagnostic and Statistical Manual of Mental Disorders [29]. The clinical diagnosis of the dementia subtype was based on international criteria for AD [30], VaD [31], dementia with Lewy body (DLB) [32], Parkinson disease dementia (PDD) [33], and other dementia [34,35]. In the CDR ≥ 1 cases, the final clinical diagnosis included six categories: possible AD, possible VaD, mixed dementia (AD + cerebrovascular disease), DLB or PDD, other dementia, and undefined (when it was not possible to fulfill any other of the previous criteria).

### Neuropathological assessment and definitions

Brain tissue was obtained within 24 h of death. One hemisphere was fixed in 4% buffered paraformaldehyde, and selected brain areas from the other hemisphere were frozen at −80°C. The following samples from the fixed hemisphere were embedded in paraffin: middle frontal gyrus, middle and superior temporal gyri, angular gyrus, superior frontal and anterior cingulate gyrus, visual cortex, hippocampal formation at the level of the lateral geniculate body, amygdala, basal ganglia at the level of the anterior commissure, thalamus, midbrain, pons, medulla oblongata, and cerebellum. Blocks were sectioned into 5-μm-thick sections. All sections were stained with hematoxylin and eosin. Immunohistochemistry with antibodies against β-amyloid (4G8, 1:10.000; Signet Pathology Systems, Dedham, Massachusetts), phosphorylated tau (PHF-1, 1:2.000; gift from Peter Davies, New York), TDP-43 (1:500, Proteintech, Chicago, Illinois), and α-synuclein (EQV-1, 1:10.000; gift from Kenji Ueda, Tokyo, Japan) were performed in selected sections [22,36,37]. Internationally accepted neuropathological criteria were used to stage and diagnose the brain pathologies [38–42]. AD-related pathology was scored using the Braak and Braak staging system for neurofibrillary pathology and the Consortium to Establish a Registry for AD (CERAD) criteria for neuritic plaque [38,39]. The new National Institutes of Health-Alzheimer Association criteria [43] were adopted by the BBBABSG in 2013. Therefore, this scale was not used in this study. A neuropathological diagnosis of AD was ascertained for individuals with Braak stage III or above and with a CERAD neuritic plaque density of moderate or frequent.

The diagnosis of argyrophilic grain disease (AGD) was based on the presence of abundant phosphorylated tau-positive grains in the CA1 sector of the hippocampus; pretangles, especially in the hippocampal CA2 sector; and oligodendrocytes with coiled bodies in the hippocampal/temporal white matter [44]. Lewy-type pathology was classified according to the Braak et al. staging scheme for PD [40]. We used the neuropathological term Lewy body disease (LBD) for all diseases associated with Lewy bodies, thereby eliminating the distinction between PD, PDD, and DLB [45]. We considered the diagnosis of LBD when Braak PD stage ≥ 3 [46].

Assessment of cerebrovascular lesions was performed macroscopically by naked eye examination and microscopically using hematoxylin and eosin stained slides in all sampled areas. The presence of small vessel disease (SVD) was evaluated according to the degree of vessel changes, localization, and extension of disease. The SVD changes included small-vessel arteriolosclerosis/atherosclerosis and lipohyalinosis [47]. SVD diagnosis required at least moderate and/or severe microvascular changes in three or more cortical regions [12]. Additionally, lacunae and large infarcts were registered by topography, stage, size, and number [12,13]. Siderocalcinosis, a vascular mineralization with an encrustation of calcium and iron in the middle layer, was evaluated in the basal ganglia and classified as present/absent [47]. Hippocampal sclerosis, defined by pyramidal cell loss and gliosis in CA1 and subiculum of the hippocampal formation, was noted and scored as present/absent [48,49]. Moreover, cerebral amyloid angiopathy (CAA) was analyzed using β-amyloid immunostaining. The localization of CAA (meningeal, gray matter, and /or white matter) as well as the severity and presence of capillary amyloid deposition was noted [50]. CAA was considered as present when it was observed widespread in the parenchyma in at least three different cortical areas.

For the current study, the diagnosis of VaD was granted to participants with either one large chronic infarct (> 1 cm) or three lacunae (< 1 cm) in any of the following strategic areas: thalamus, frontocingular cortex, basal forebrain and caudate, medial temporal area, or angular gyrus [12]. SVD only was not enough for granting a VaD diagnosis. Neuropathological diagnoses were made blinded to clinical status.

Immunohistochemistry to detect TDP-43 (1:500, Proteintech, Chicago, Illinois), in at least hippocampal formation and amygdala, was introduced in our routine in 2012, and previous cases are being reassessed (to date, a total of 347 cases underwent TDP-43 assessment). Furthermore, all cases with an undetermined neuropathological diagnosis or a clinical diagnosis of frontotemporal dementia or primary progressive aphasia underwent extensive immunohistochemistry for TDP-43 [5].

### Apolipoprotein E (APOE) genotyping

When DNA was available, APOE genotypes (single-nucleotide polymorphisms rs429358 and rs7412) were determined by allele-specific amplification real-time PCR assays, in duplicates, as previously described [51].

### Statistical analysis

Study design and statistical analyses for this study were planned 2 y after obtaining the data. We compared participants in the three categories of dementia status (CDR = 0: no dementia; CDR = 0.5: questionable dementia; and CDR ≥ 1: dementia) regarding clinical, APOE allele ε4, and neuropathological variables using one-way ANOVA or Kruskal-Wallis when variables were quantitative and using chi-square or Fisher’s exact tests when variables were categorical. We had first planned to include in the multivariate model only the variables that were associated with dementia status in univariate analyses. However, following peer review request, we included all neuropathological variables measured in this study in a multivariate ordinal logistic regression model that had the three categories of cognitive status (normal, questionable dementia, and dementia) as the dependent variable, since these variables have been associated with higher risk of dementia in several studies [9,10,19,44,47]. The ordinal logistic model was adjusted for age, sex, and education. The variables associated with dementia status at the 0.05 alpha level were selected. We used the coefficients from the ordinal logistic regression model with the selected variables to develop a neuropathological comorbidity (NPC) score. Since each neuropathological lesion was associated with different odds of dementia, points were assigned to each neuropathological variable by dividing each coefficient by the lowest coefficient (i.e., siderocalcinosis) and rounding up or down to the nearest integer [52,53]. An NPC score was assigned for each participant by adding the points for each neuropathological variable present. We then investigated the association between NPC scores with three different outcomes (CDR-SOB, IQCODE, and NPI total score), using linear regression models adjusted for age, sex, and education. Additionally, we used linear regression models to investigate whether there was an interaction between the NPC score, excluding Braak neurofibrillary tangle (NFT) score, and cognitive scores to answer the question of whether these non-AD-related lesions have a synergic or additive effect to Braak NFT score on cognitive symptoms [20,54,55].

We described the absolute and relative frequencies of the neuropathological diagnoses in all participants and also stratified by dementia status. We then calculated the sensitivity (the number of true positives divided by the number of true positives plus false negatives), specificity (the number of true negatives divided by the number of true negatives plus positives), and accuracy (the number of true positives plus true negatives divided by the total number of participants) of the clinical diagnosis of dementia subtypes compared to the neuropathological diagnoses, which was considered the gold standard. We used Stata 13.0 (StataCorp, College Station, Texas) to perform the statistical analyses. The alpha level for all statistical tests was set at 0.05 level in two-tailed tests.

## Results

### Participants

Between January 2004 and December 2014, 650,837 people (50% male) died in the city of São Paulo with a mean age at death of 73±12 y [56]. During this period, 104,385 persons (16% of the total number of deaths) were autopsied in SPAS. The mean age of the 1,092 participants of this study was 74±12 y, and 49% were male, both similar to the death data from São Paulo city. Moreover, 69% were identified by next-of-kin as white, 11% black, 18% brown, and 2% were from other races. The mean years of education was 4.2±3.7 y. Cardiovascular risk factors and diseases were frequent, as 66% reportedly had hypertension, 27% diabetes, and 24% had coronary artery disease. Regarding cognitive outcomes, 61% had CDR = 0, the mean IQCODE score was 3.41±0.67, and the median NPI score was 5. APOE genotype was available for 524 participants. In this subsample, 155 individuals had at least one APOE allele ε4 (S1 and S2 Tables).

Regarding the dementia status, participants with dementia were older, predominantly female, and had a lower education level (Table 1). Interestingly, the dementia group had a lower prevalence of some cardiovascular risk factors and diseases, like hypertension, coronary artery disease, heart failure, smoking, and alcohol use (Table 1). As expected, IQCODE scores were higher and neuropsychiatric symptoms were more common in demented participants (CDR ≥ 1). APOE allele ε4 was more common in the dementia group (Table 1).

**Table 1:** Sociodemographics, clinical variables, and APOE genotype, according to dementia status (*n* = 1,092).

	No dementia, CDR = 0 (*n* = 665)	Questionable dementia, CDR = 0.5 (*n* = 123)	Dementia, CDR ≥ 1 (*n* = 304)	*p*-Value
Age (years), mean (SD)*	71.7 (12.0)	74.1 (11.2)	79.5 (9.8)	<0.001
**Male, %**^‡^	52.9	47.1	39.5	<0.001
**Race, %**^†^				0.15
*White*	69.3	65.0	71.3	
*Black*	10.7	9.8	11.9	
*Brown*	17.3	24.4	16.2	
*Other*	2.7	0.8	0.6	
**Education (years), mean (SD)**^§^	4.7 (3.8)	3.5 (2.7)	3.3 (3.5)	<0.001
**Hypertension, %**^‡^	67.9	72.3	58.0	0.003
**Diabetes, %**^‡^	25.9	30.0	28.0	0.57
**Coronary artery disease, %**^‡^	26.7	27.4	18.2	0.02
**Heart failure, %**^‡^	17.5	23.0	13.4	0.06
**Arrhythmia, %**^‡^	9.0	14.1	8.3	0.23
**Dyslipidemia, %**^‡^	9.0	14.3	6.7	0.05
**Stroke, %**^‡^	10.5	19.8	26.9	<0.001
Body mass index (kg/m^2^), mean (SD)*	23.9 (4.8)	23.2 (4.0)	21.2 (4.5)	<0.001
**Smoking, %**^‡^				<0.001
*Never*	60.3	61.6	68.4	
*Current*	31.1	27.3	14.1	
*Previous*	8.6	11.1	17.5	
**Alcohol use, %**^‡^				<0.001
*Never*	85.1	82.8	80.0	
*Current*	10.5	10.1	6.4	
*Previous*	4.4	7.1	13.6	
IQCODE, mean (SD)*	3.01 (0.04)	3.26 (0.17)	4.34 (0.60)	<0.001
NPI, mean (SD)*	6.5 (10.2)	10.3 (13.7)	24.4 (21.4)	<0.001
**APOE genotype, %**^†^^,^ ^┐^				0.005
*One allele ε4*	13.4	8.1	13.2	
*Two alleles ε4*	0.6	0.8	3.6	

APOE, apolipoprotein E; CDR, Clinical Dementia Rating; IQCODE, Informant Questionnaire on Cognitive Decline in the Elderly; NPI, Neuropsychiatric Inventory; SD, standard deviation.

*One-way ANOVA;

^§^Kruskal-Wallis test;

^‡^Chi-square test;

^†^Fisher’s exact test;

^┐^Data available for 524 participants

### Neuropathological diagnoses and cognitive status

Forty-four percent of the sample (*n* = 480), regardless of cognitive status, had enough lesions to meet criteria for a neuropathological diagnosis (S1 Fig). Among these 480 participants, mixed neuropathology was present in 20% of the sample. The most common neuropathological diagnosis was AD alone or in combination with other neuropathological diagnoses, which was present in 240 individuals (50% of the 480 participants with neuropathological diagnosis). The diagnosis of VaD was ascertained in 170 individuals (35%), LBD in 87 (18%), and other neuropathological diagnoses in 77 (16%). Within the category “Other,” we grouped neuropathological diagnoses with low frequencies, including four instances of FTLD-TDP, eight of FTLD with tau inclusions (two cases of progressive supranuclear palsy, one of corticobasal degeneration, one of Pick disease, and four unspecified tauopathies) [5], three pure CAA cases, five instances of tangle-only dementia [57], one case of multiple system atrophy, and one diagnosis of hippocampal sclerosis of aging [17].

**Fig 1:**
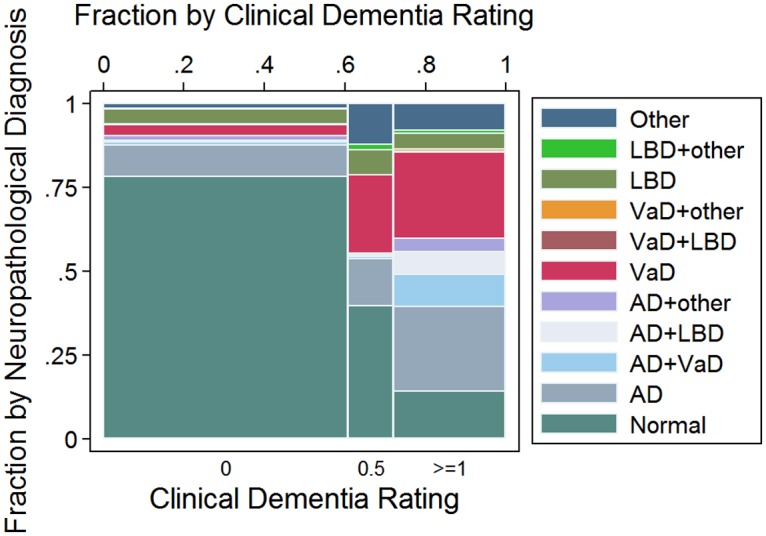
Mosaic plot showing the relationship between neuropathological classification and dementia status according to the Clinical Dementia Rating (CDR) scale: CDR = 0: No dementia; CDR = 0.5: Questionable dementia; And CDR ≥ 1: Dementia. AD, Alzheimer disease; LBD, Lewy body disease; VaD, vascular disease.

Fig 1 shows the neuropathological diagnosis by dementia status. Among the 665 participants with normal cognition (CDR = 0), 145 (22%) met the criteria for a neuropathological diagnosis, with AD as the most common diagnosis (*n* = 82; 57% of the CDR = 0 individuals with a neuropathological diagnosis). Among the 123 participants with questionable dementia (CDR = 0.5), 74 (60%) met the criteria for a neuropathological diagnosis, with VaD as the most common one (*n* = 30; 41%). Finally, among the 304 participants with dementia (CDR ≥ 1), 261 (86%) met the criteria for at least one neuropathological diagnosis. AD alone or in combination with other neuropathological lesions was present in 139 CDR ≥ 1 participants (53%), VaD in 110 (42%), and LBD in 39 (15%). Forty-three CDR ≥ 1 participants (14%) did not have enough neuropathological lesions to fulfill criteria for any neuropathological diagnoses.

### Association between neuropathological lesions and dementia status

**Fig 2:**
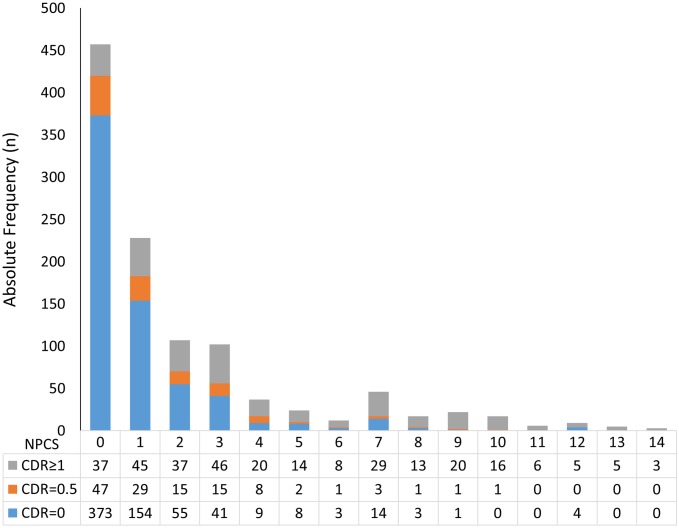
Number of participants in each stratum of the Neuropathological Comorbidity Score (NPCS) according to dementia status defined by the CDR scale (CDR = 0: No dementia; CDR = 0.5: Questionable dementia; CDR ≥ 1: Dementia).

**Fig 3:**
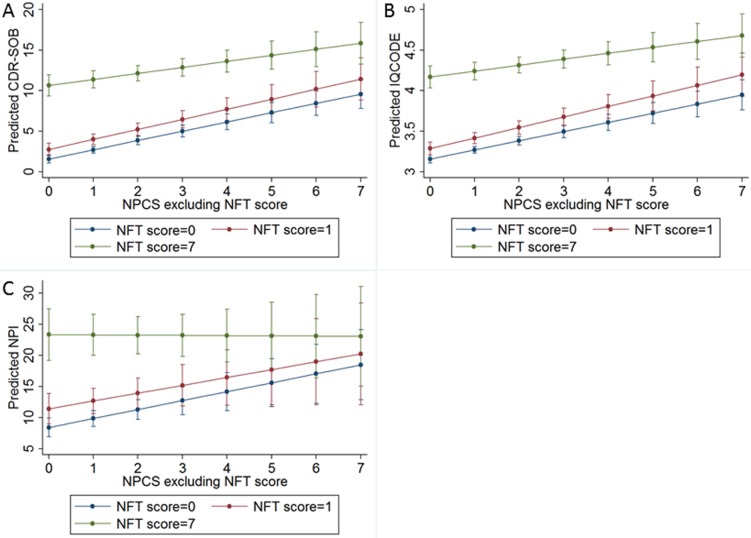
Predicted values of (A) the CDR Sum of Boxes (CDR-SOB), (B) the Informant Questionnaire on Cognitive Decline in the Elderly (IQCODE), and (C) the Neuropsychiatric Inventory (NPI) considering the Neuropathological Comorbidity Score (NPCS) without Alzheimer disease pathology for participants with Neurofibrillary Tangle (NFT) score = 0 (Braak NFT stage = 0–II: Blue line), NFT score = 1 (Braak NFT stage = III–IV: Red line), and NFT score = 7 (Braak NFT stage = V–VI: Green line). Predicted values of cognitive and neuropsychiatric outcomes were obtained by multivariate linear models adjusted for age, sex, and education.

In univariate analyses, all neuropathological lesions, except AGD, were associated with dementia (*p* < 0.001, except for CAA with *p* = 0.004) (Table 2). A multivariable ordinal logistic model including all neuropathological variables indicated that Braak NFT stage, hippocampal sclerosis, lacunar infarcts, hyaline arteriolosclerosis, siderocalcinosis, and Braak LBD stage were associated with dementia status at the 0.05 level (S3 Table). We used these variables to develop our NPC score (Table 3). Fig 2 shows the number of individuals at each strata of the NPC score, according to three categories of dementia status. Higher NPC scores were associated with higher CDR-SOB (β = 1.15; 95% CI 1.04–1.27; *p* < 0.001), IQCODE (β = 0.12; 95% CI 0.11–0.14; *p* < 0.001), and NPI (β = 1.50; 95% CI 1.14–1.85; *p* < 0.001) scores (S4 Table). Since the Braak NFT score showed a disproportionately higher weight than the other variables in the NPC score, we also tested the association between all the other elements of the NPC scores together and cognitive and neuropsychiatric outcomes. NPC scores devoid of the Braak stage element remained associated with worse CDR-SOB, IQCODE, and NPI scores (S4 Table). Next, we tested the interaction between the Braak NFT score in isolation and all the other elements of the NPC scores as a group on cognitive and neuropsychiatric outcomes in models adjusted for age, sex, and education. We failed to find a multiplicative interaction between Braak NFT scores and the other types of neuropathological lesions (S4 Table and Fig 3).

**Table 2:** Frequency of neuropathological lesions per dementia status (*n* = 1,092).

	No dementia, CDR = 0 (*n* = 665)	Questionable dementia, CDR = 0.5 (*n* = 123)	Dementia, CDR ≥ 1 (*n* = 304)	*p*-Value
**CERAD neuritic plaque score, *n* (%)**^‡^				<0.001
***None or Scarce***	464 (69.7)	79 (64.2)	126 (41.5)	
***Moderate***	91 (13.7)	23 (18.7)	36 (11.8)	
***Frequent***	110 (16.6)	21 (17.1)	142 (46.7)	
**Braak NFT Stage, *n* (%)**^†^				<0.001
***0–II***	499 (75.0)	82 (66.7)	120 (39.5)	
***III–IV***	145 (21.8)	36 (29.3)	90 (29.6)	
***V–VI***	21 (3.2)	5 (4.0)	94 (30.9)	
**Hippocampal sclerosis, *n* (%)**^†^	8 (1.2)	4 (3.2)	18 (5.9)	<0.001
**Lacunar infarcts, *n* (%)**^‡^	34 (5.1)	20 (16.4)	77 (25.6)	<0.001
**Hyaline arteriolosclerosis, *n* (%)**^‡^	58 (8.8)	14 (11.5)	86 (28.3)	<0.001
**Cerebral amyloid angiopathy, *n* (%)**^‡^	19 (2.9)	6 (4.9)	23 (7.6)	0.004
**Siderocalcinosis, *n* (%)**^‡^	92 (13.9)	23 (18.9)	74 (24.4)	<0.001
**Argyrophilic grain disease**	116 (17.5)	18 (14.6)	44 (14.9)	0.49
**Lewy body disease (Braak Stage), *n* (%)**^†^				<0.001
***0***	612 (92.0)	111 (90.2)	257 (84.5)	
***I–III***	32 (4.8)	7 (5.7)	10 (3.3)	
***IV–VI***	21 (3.2)	5 (4.1)	37 (12.2)	
**TDP-43, *n* (%)**^†^^§^	27 (9.0)	3 (18.8)	9 (30.0)	0.002

CERAD, Consortium to Establish a Registry for Alzheimer's Disease; NFT, neurofibrillary tangle.

^‡^Chi-square test;

^†^Fisher’s exact test;

^§^Data available for 347 participants

**Table 3:** Neuropathological lesions that were independently associated with dementia status in multivariate ordinal logistic regression (*n* = 1,092).

	OR (95% CI)*	Points
**Braak NFT Stage**		
***0–II***	1 (reference)	0
***III–IV***	1.81 (1.31–2.49)	1
***V–VI***	10.88 (6.54–18.10)	7
**Hippocampal sclerosis**	2.80 (1.23–6.37)	2
**Lacunar infarcts**	4.57 (3.06–6.82)	3
**Hyaline arteriolosclerosis**	2.36 (1.61–3.47)	2
**Siderocalcinosis**	1.55 (1.10–2.19)	1
**Lewy body disease (Braak Stage)**		
***0–III***	1 (reference)	0
***IV–VI***	3.40 (1.94–5.97)	2

OR, odds ratio. Multivariable ordinal logistic regression model, adjusted for age, sex, education, and other neuropathological lesions in the table; the dependent variable was three categories of the CDR score: CDR = 0 (reference), CDR = 0.5, and CDR ≥ 1.

### Clinicopathological correlations

Table 4 shows the correlation between clinical and neuropathological diagnoses in participants with CDR ≥ 1. According to the clinical consensus, 45% of the participants with dementia met clinical diagnosis for probable or possible AD, 20% met criteria for AD plus cerebrovascular disease, and 17% met criteria for VaD. Considering the neuropathological diagnosis as the gold standard, the sensitivity of the clinical diagnosis for AD (pure AD plus AD with cerebrovascular disease) was 83%, the specificity was 47%, and the accuracy was 61%. The clinical diagnosis of possible VaD (VaD alone or in combination with AD) had a sensitivity of 47%, specificity of 70%, and accuracy of 61%, while the clinical diagnosis of LBD or dementia related to PD had a sensitivity of 18%, specificity of 97%, and accuracy of 86% (Table 4).

**Table 4:** Comparison between clinical and neuropathological diagnoses among participants with dementia (CDR ≥ 1) (*n* = 287).

Neuropathological diagnosis	Clinical diagnosis						Total (%)
	AD	VaD	AD+CVD	LBD/PD	Other	Undefined	
**AD**	40	8	14	4	3	2	71 (24.7)
**AD + VaD**	16	3	8	1	1	0	29 (10.1)
**AD + LBD**	11	1	4	1	1	2	20 (7.0)
**AD + other**	5	1	5	0	0	1	12 (4.2)
**VaD**	26	22	17	1	4	4	74 (25.8)
**VaD + LBD**	0	0	0	0	0	1	1 (0.3)
**VaD + other**	2	0	0	0	0	0	2 (0.7)
**LBD**	5	2	1	5	1	0	14 (4.9)
**LBD + other**	1	0	1	1	0	0	3 (1.0)
**Other**	8	6	3	1	2	3	23 (8.0)
**Normal**	14	5	4	1	9	5	38 (13.2)
**Total (%)**	128 (44.6)	48 (16.7)	57 (19.9)	15 (5.2)	21 (7.3)	18 (6.3)	287 (100)*
**Sensitivity**	82.8%^†^	47.2%^‡^	–	18.4%^┐^	–	–	
**Specificity**	47.1%^†^	69.6%^‡^	–	96.8%^┐^	–	–	
**Accuracy**	61.3%^†^	61.3%^‡^		86.4%^┐^			

AD, Alzheimer disease; CVD, cerebrovascular disease; LBD, Lewy body disease; PD, Parkinson disease; VaD, vascular dementia.

*Seventeen participants had missing information for the clinical diagnosis of dementia subtype;

^†^Clinical diagnosis of AD plus AD+CVD compared to neuropathological diagnosis of AD+VaD plus AD+LBD plus AD+other;

^‡^Clinical diagnosis of VaD plus AD+CVD compared to neuropathological diagnosis of AD+VaD plus VaD+LBD plus VaD+other;

^┐^Clinical diagnosis of LBD/PD compared to neuropathological diagnosis of AD+LBD plus VaD+LBD plus LBD+other

## Discussion

Here we described neuropathological findings and clinical and neuropathological variables associated with cognitive impairment in a community-based sample of 1,092 older adults from a LMIC with broad educational attainment and admixed race background, a population profile much different compared to the samples enrolled in other neuropathologic studies conducted in developed countries. The main findings of this study were as follows:

1. Neurodegenerative and cerebrovascular lesions are common in older adults from a LMIC, similar to data from HICs. Furthermore, almost one-fourth of cognitively normal individuals met the criteria for a neuropathological diagnosis, and these numbers increase progressively in participants with questionable dementia and dementia, respectively. AD was the most common neuropathological diagnosis in the CDR = 0 and CDR ≥ 1 groups, followed by VaD. In fact, VaD was the most common diagnosis in the CDR = 0.5 group. Finally, a small fraction of individuals lack a neuropathological diagnosis to explain the cognitive impairment.
2. NFT burden, hippocampal sclerosis, lacunar infarcts, hyaline atherosclerosis, siderocalcinosis, and LBD were independently associated with cognitive impairment. NFT burden was the main driver of the association between higher NPC scores and worse cognitive and neuropsychiatric outcomes. Overlapping neuropathological lesions had an additive rather than a multiplicative impact on cognition.
3. The clinical criteria for LBD presented the highest specificity and accuracy, although the sensibility was very poor. Both clinical criteria for AD and VaD showed a moderate accuracy.

In our total sample, a large percentage of individuals met the criteria for a neuropathological diagnosis. Out of them, 50% met the criteria for AD, including AD associated with other diseases. These numbers are in accordance with other clinicopathological studies, in which the AD prevalence ranged from 19% to 65% [58–61]. In contrast, the prevalence of pathologically confirmed VaD alone or in combination with other pathology (35%) was on the higher end compared to other reported prevalence rates, which ranged from 8% to 45% [58–60,62]. Interestingly, these high frequencies of VaD corroborate our previous findings from a smaller sample of the same population [12]. The high prevalence of VaD alone or in combination with AD found in the present study might reflect the limited access of the study population to basic health care and, consequently, poor control of cerebrovascular risk factors. In addition, other factors might have contributed to the high proportion of VaD. The Brazilian population is highly mixed genetically, mainly due to historical waves of immigration from Africa, Europe, and Asia. We have shown before that African and Japanese ancestry may modify the risk for specific neurodegenerative lesions [36,63]. Quantitative studies are necessary to investigate if the same is true for cerebrovascular lesions. Nevertheless, other biases could explain the high prevalence of VaD in our sample. The use of different neuropathological criteria could also play a role [64,65], and although the BBBASG follows internationally accepted criteria and the neuropathologists (LTG and RDR) have broad experience in international centers, a universally accepted neuropathological criteria for VaD has yet to be established, and each center has adopted its own criteria [66,67]. Even the nature of the cerebrovascular lesions believed to cause cognitive decline varies considerably [47]. We used stringent criteria for VaD. Had isolated—but widespread—SVD been considered part of our criteria, the proportion of VaD diagnosed in our series would have been even greater (from 35% of the total number of cases fulfilling the criteria for a neuropathology diagnosis to 49%). The proportion of individuals with LBD (18%), including PD and DLB alone or in combination with other pathology, was similar to other studies [68,69] but lower than studies with older individuals [70].

When grouping the participants by cognitive status, we found that more than 20% of CDR = 0 individuals met the criteria for one or more neuropathological diagnoses. Among these individuals with CDR = 0 and neuropathological diagnoses, AD was the most common diagnosis (57%), which is in line with rates reported previously [71–73]. These numbers go in accordance with other studies in which the rates of neuropathological AD in the nondemented groups were 7% [74] and 22% [75], respectively, using the CERAD criteria plus Braak NFT stage, compared with 33% [74] and 55% [75], respectively, using the CERAD criteria alone. Had we considered only the CERAD scores, the prevalence of AD in the CDR = 0 group with neuropathological diagnoses would be 69%.

This asymptomatic sample with high neuropathological burden is one of the largest reported to date, and it offers a unique opportunity to investigate factors associated with cognitive reserve. For instance, participants without dementia had higher education attainment than participants with CDR ≥ 0.5 in this study. In fact, we have shown in one of our previous smaller studies that even a few years of education was associated with better cognitive abilities [13].

Sixty percent of participants with CDR = 0.5 met the criteria for at least one neuropathological diagnosis. VaD was the most common diagnosis in this subgroup, highlighting that cerebrovascular lesions may lead to milder cognitive impairment. Out of the 46 CDR = 0.5 individuals who did not meet criteria for a neuropathological diagnosis, two had major depression, one had schizophrenia, and one was a heavy alcohol user. Also, a large portion of these individuals had some degree of neurodegenerative lesions that could have impacted cognition. Future work quantifying these lesions for clinicopathological correlation may help to shed light on this question.

Among participants with CDR ≥ 1, AD alone or in combination accounted for 53% of the neuropathological diagnoses, followed closely by VaD alone or in combination (42%). The prevalence of AD is also in line with previous large autopsy studies, although the VaD prevalence is close to the higher rate of VaD reported [59]. Forty-three participants did not have enough cerebral lesions to ascertain a neuropathological diagnosis. Among them, 10 had clinical diseases that could explain the cognitive impairment: three had major depression, four were heavy alcohol users, one could have had cognitive symptoms secondary to cancer, and two had schizophrenia—although schizophrenia is an exclusion criterion for the diagnosis of dementia, these individuals presented cognitive deficiency, suggesting dementia; thus, they were included in the study. Dementia without a clear explanation has also been reported elsewhere, including in a series of 128 demented patients, in which 16% did not have enough brain lesions to explain the symptoms [76].

Our data corroborate that AD-related tau pathology (NFT burden) and cerebrovascular lesions (lacunar infarcts and SVD) were independently associated with cognitive impairment. Particularly, the presence of Braak NFT stages V/ VI was related to much higher odds of dementia in our sample and in other studies [9,18,74,77–81]. Moreover, hippocampal sclerosis, siderocalcinosis, and LBD were also independently related to moderately higher odds of dementia [9,19,47,82]. By using a NPC score and analyzing it against CDR-SOB, IQCODE, and NPI, we showed that neuropathological comorbidity is related to increased risk of dementia and the severity of neuropsychological symptoms. These results are in line with other cohorts from HICs [9,10,19,20,83]. One open question refers to whether neuropathological comorbidity results in a synergistic effect of worsening cognition in humans, as has been shown in animal models [55]. Our data failed to show a multiplicative effect of neuropathological comorbidity with cognition. Studies in humans assigning a weighted score to different neuropathological lesions are necessary to verify our findings.

Although beta-amyloid plaques are considered by many to be the main neuropathological hallmark of AD, beta-amyloid plaque burden did not affect cognitive or neuropsychiatric scores in this series or in other community-based studies [74,75,84]. Likewise, AGD, a common age-related tauopathy [44], failed to worsen cognitive scores. Other studies suggest that AGD can actually represent a protective factor against AD spread [85].

We detected relatively high sensitivity and low specificity for the clinical diagnosis of AD, while there was a high specificity with very low sensitivity for DLB and Parkinson disease dementia. The same findings were reported in a study that involved 31 United States academic medical centers as well as in another Swedish study [69,86]. We found low sensitivity and moderate specificity for the diagnosis of VaD. Low sensitivity of the clinical criteria for VaD has been found in other community-based studies [86,87], particularly those using the National Institute of Neurological Disorders and Stroke–Association Internationale pour la Recherche et l'Enseignement en Neurosciences (NINDS-AIREN) criteria [88,89]. Additionally, we have adopted conservative neuropathological criteria for VaD, which could have contributed to the low sensitivity of the clinical criteria.

We found several associations between demographics and clinical variables and cognitive impairment. Older age, being a woman, lower educational attainment, and a previous diagnosis of stroke were associated with dementia status in our study and are in line with results from other studies [61,90,91]. Hypertension, coronary artery disease, higher body mass index, currently smoking, and alcohol use were more common among participants without dementia. Although paradoxal, the inverse associations between some risk factors and dementia in late life has been described before [92,93] and may reflect a secondary effect associated with dementia (e.g., low body mass index [BMI] due to appetite loss) or survival bias [94].

### Strengths and limitations of the study

Our study has several advantages. We presented clinical and neuropathological data from a large sample of individuals who had a low educational level (mean education of 4 y) and were of admixed race. Previous large studies included mainly white or Asian participants with high education attainment (mean education of 16 y in most studies) [9,78]. We also collected comprehensive clinical and neuropathological data that allowed us to develop the NPC score and investigate its association with cognitive and neuropsychiatric scales. Moreover, we investigated the association between clinical symptoms and AGD, which has not been fully explored in other series [44]. In addition, the BBBABSG is a community-based autopsy study, which allows for the collection of a large number of brains from individuals with normal cognition and also an opportunity to study participants with dementia of unknown etiology.

However, our results should be examined considering the study limitations. We did not follow participants during life, and clinical variables were evaluated postmortem through an interview with an informant. To increase the reliability of these data, we included only participants who had at least weekly contact with the informant and excluded individuals when the informant provided conflicting information during the clinical interview. In addition, we have shown that the postmortem cognitive evaluation had a sensitivity of 87% and specificity of 84% for the clinical diagnosis of dementia [23]. Second, the neuropathological criteria for the diagnosis of VaD have not been standardized through different research centers [47]. We used a conservative neuropathological criterion for VaD. In addition, some recent neuropathological diagnoses, such as chronic traumatic encephalopathy or TDP-43 deposition [95], were not systematically investigated in our sample. Although we assessed all individuals with an undetermined neuropathological diagnosis or a clinical diagnosis of frontotemporal dementia or primary progressive aphasia to rule out the possibility of FTLD-TDP, we only completed TDP-43 assessment for 347 participants. Despite the fact that TDP-43 inclusions are found in a considerable percentage of brains of cognitively normal elderly, evidence shows that TDP-43 inclusions in limbic structures may worsen the cognition in the context of AD [95,96]. Finally, our sample is not representative of all deaths that occurred in São Paulo during the study period. SPAS receives people who die from natural causes, and cardiac arrests are common in this population; therefore, we might have over-represented individuals with cardiovascular disease and that might have increased the frequency of cerebrovascular lesions in our sample (participants with and without dementia), but it should be emphasized that cerebrovascular lesions were much more frequent in the group with dementia. On the other hand, individuals with macroscopically detectable acute brain infarctions, hemorrhages, or trauma were underrepresented in the BBBABSG, as an immediate examination was required for the completion of the death certificate, and this fact might have decreased the frequency of cerebrovascular lesions in our sample. Unfortunately, selection bias has been found in most population-based studies on dementia. Almost half of the first 209 individuals submitted to autopsy in the CFAS study had dementia, a much higher proportion than the one found in the general population [74], whereas the Honolulu-Asia Aging study only recruited Americans with Japanese ethnicity [97] and the Nun study only included white women [98]. The Religious Order study mainly included individuals who identified as white, and the mean age of death was higher than that of the general population [77,99]. Average older age at death was also present in other studies [20,100–102].

In conclusion, we described the association of neuropathological lesions and cognitive and neuropsychiatric outcomes in a unique sample of Brazilian individuals with low education attainment and admixed race. In concordance with previous studies, NFT deposition and high comorbidity neuropathological scores were highly associated with dementia status. We also found a higher frequency of cerebrovascular lesions than previously described. Future clinicopathological studies with populations in LMICs are important to confirm our findings.

## Supporting information

S1 Checklist STROBE guidelines. (DOC)

S1 Fig Neuropathological classification of participants (*n* = 1,092). AD, Alzheimer disease; LBD, Lewy body disease; VaD, vascular disease.
(TIF)

S1 Table Characteristics of the sample (*n* = 1,092). (DOCX)

S2 Table Frequency of neuropsychiatric symptoms according to dementia status (*n* = 1,092). (DOCX)

S3 Table Association between neuropathological lesions and dementia status (*n* = 1,092). (DOCX)

S4 Table Association between NPC score and cognitive outcomes (*n* = 1,092). (DOCX)

## Abbreviations

AD: Alzheimer disease
AGD: argyrophilic grain disease
APOE: apolipoprotein E
BBBABSG: Brain Bank of the Brazilian Aging Brain Study Group
BMI: body mass index
CAA: cerebral amyloid angiopathy
CDR: Clinical Dementia Rating
CDR-SOB: CDR sum of boxes
CERAD: Consortium to Establish a Registry for Alzheimer's Disease
DLB: dementia with Lewy body
FTLD: frontotemporal lobar degeneration
FTLD-TDP: FTLD with TDP-43 inclusions
HIC: high-income country
IQCODE: Informant Questionnaire on Cognitive Decline in the Elderly
LBD: Lewy body disease
LMIC: low- and middle-income country
NFT: neurofibrillary tangle
NINDS-AIREN: National Institute of Neurological Disorders and Stroke–Association Internationale pour la Recherche et l'Enseignement en Neurosciences
NPC: neuropathological comorbidity
NPI: Neuropsychiatric Inventory
SPAS: São Paulo Autopsy Service
STROBE: Strengthening the Reporting of Observational Studies in Epidemiology
SVD: small vessel disease
TDP-43: transactivation response DNA-binding protein of 43 kDa
VaD: vascular dementia
