# Supplementary figures and images for: Neuropathological diagnoses and clinical correlates in older adults in Brazil: A cross-sectional study
Source: PLoS Med. 2017 Mar 28;14(3):e1002267. doi: 10.1371/journal.pmed.1002267 (PMC5369698; doi:10.1371/journal.pmed.1002267)

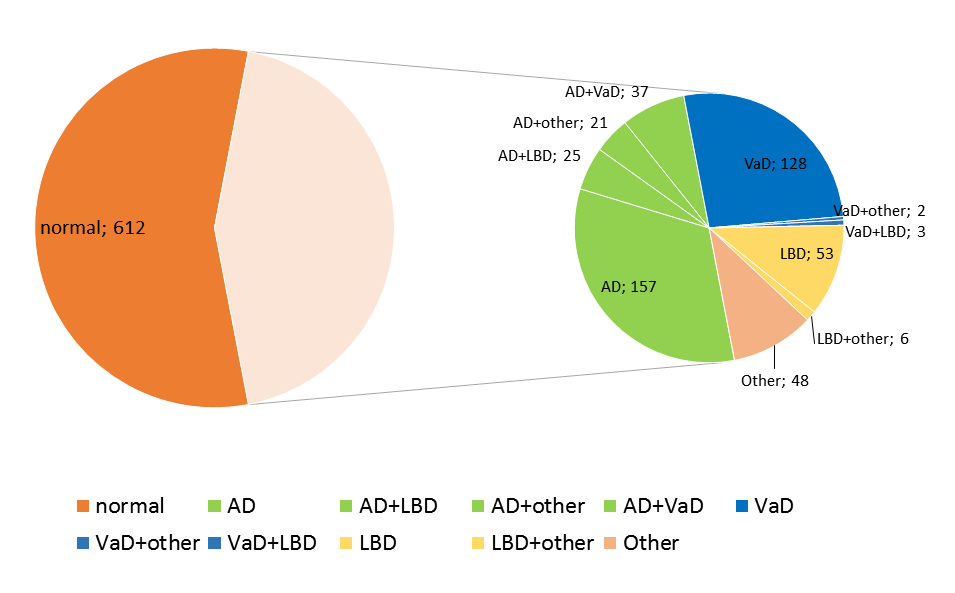

Supplement: S1 Fig — AD, Alzheimer disease; LBD, Lewy body disease; VaD, vascular disease. (TIF) [file pmed.1002267.s002.tif]
