# Supplementary material for: Neuropathological diagnoses and clinical correlates in older adults in Brazil: A cross-sectional study
Source: PLoS Med. 2017 Mar 28;14(3):e1002267. doi: 10.1371/journal.pmed.1002267 (PMC5369698; doi:10.1371/journal.pmed.1002267)
Supplement: S1 Table — (DOCX) [file pmed.1002267.s003.docx]

S1 Table. Characteristics of the sample (n=1,092)

| Variables | Mean (SD) or % |
| --- | --- |
| Age (years) | 74.1 (11.8) |
| Male | 48.5% |
| Race  *. White*  *. Black*  *. Brown*  *. Asian* | 69.4  10.9  17.8  1.9 |
| Education (years) | 4.2 (3.7) range 0-25 |
| Socioeconomic status  *. High*  *. Middle*  *. Low* | 24.9  40.5  34.7 |
| Hypertension | 65.6 |
| Diabetes | 26.9 |
| Coronary artery disease | 24.5 |
| Heart failure | 17.0 |
| Arrhythmia | 9.3 |
| Dyslipidemia | 8.9 |
| Stroke | 16.1 |
| Body mass index (kg/m^2^) | 23.2 (4.8) |
| Smoking  *. Never*  *. Current*  *. Previous* | 62.6  26.1  11.3 |
| Alcohol use  *. Never*  *. Current*  *. Previous* | 83.5  9.4  7.1 |
| Clinical Dementia Rating (CDR)  *. 0*  *. 0.5*  *. 1*  *. 2*  *. 3* | 60.9  11.3  7.7  6.0  14.2 |
| CDR Sum of Boxes | 3.95 (6.36) range 0-18 |
| IQCODE | 3.41 (0.67) |
| NPI | 11.9 (16.5) range 0-116 |
| Hallucinations | 15.7 |
| Delusions | 10.7 |
| Agitation/Aggression | 21.7 |
| Dysphoria/Depression | 26.3 |
| Anxiety | 25.9 |
| Irritability | 17.8 |
| Disinhibition | 8.8 |
| Euphoria | 4.6 |
| Apathy | 20.7 |
| Aberrant motor behavior | 9.9 |
| Sleep and night-time behavior change | 24.4 |
| Appetite and eating change | 33.6 |
| Post-mortem interval (hours) | 14.4 (4.1) |
| Daily contact with the deceased | 80.0 |
| APOE genotype*  *. 22*  *. 23*  *. 24*  *. 33*  *. 34*  *. 44* | 1.0  9.3  1.5  60.1  25.0  3.1 |

IQCODE: Informant Questionnaire on Cognitive Decline in the Elderly; NPI: Neuropsychiatric Inventory

*missing data for 568 participants
