# Supplementary material for: Neuropathological diagnoses and clinical correlates in older adults in Brazil: A cross-sectional study
Source: PLoS Med. 2017 Mar 28;14(3):e1002267. doi: 10.1371/journal.pmed.1002267 (PMC5369698; doi:10.1371/journal.pmed.1002267)
Supplement: S2 Table — (DOCX) [file pmed.1002267.s004.docx]

S2 Table. Frequency of neuropsychiatric symptoms according to dementia status (n=1,092)

|  | No dementia  CDR=0  (n=665) | Questionable Dementia  CDR=0.5  (n=123) | Dementia  CDR=≥1  (n=304) | p |
| --- | --- | --- | --- | --- |
|  |  |  |  |  |
| Hallucinations, %^‡^ | 5.3 | 11.4 | 40.1 | <0.0001 |
| Delusions, %^‡^ | 4.1 | 6.5 | 27.0 | <0.0001 |
| Agitation/Aggression, %^‡^ | 13.1 | 15.4 | 43.1 | <0.0001 |
| Dysphoria/Depressive symptoms, %^‡^ | 19.6 | 31.7 | 38.8 | <0.0001 |
| Anxiety, %^‡^ | 24.2 | 26.0 | 29.6 | 0.21 |
| Irritability, %^‡^ | 12.6 | 21.1 | 27.3 | <0.0001 |
| Disinhibition, %^‡^ | 3.0 | 6.5 | 22.4 | <0.0001 |
| Euphoria, %^†^ | 3.6 | 1.6 | 7.9 | 0.004 |
| Apathy, %^‡^ | 12.2 | 16.3 | 41.1 | <0.0001 |
| Aberrant motor behavior, %^‡^ | 4.2 | 5.7 | 24.0 | <0.0001 |
| Sleep and night-time behavior change, %^‡^ | 19.4 | 19.5 | 37.5 | <0.0001 |
| Appetite and eating change, %^‡^ | 28.9 | 22.8 | 48.4 | <0.0001 |
