# Supplementary material for: Neuropathological diagnoses and clinical correlates in older adults in Brazil: A cross-sectional study
Source: PLoS Med. 2017 Mar 28;14(3):e1002267. doi: 10.1371/journal.pmed.1002267 (PMC5369698; doi:10.1371/journal.pmed.1002267)
Supplement: S3 Table — (DOCX) [file pmed.1002267.s005.docx]

S3 Table. Association between neuropathological lesions and dementia status (n=1,092)

|  | OR (95% CI)* | p |
| --- | --- | --- |
| CERAD Neuritic Plaque Score  *. None or Mild*  *. Moderate*  *. Severe* | 1.0 (reference)  1.03 (0.69-1.54)  1.06 (0.68-1.65) | -  0.87  0.80 |
| Braak NFT Stage  *. 0-II*  *. III-IV*  *. V-VI* | 1.0 (reference)  1.94 (1.34-2.81)  10.42 (5.56-19.54) | -  <0.0001  <0.0001 |
| Hippocampal sclerosis | 2.55 (1.11-5.85) | 0.03 |
| Lacunar infarcts | 4.54 (3.04-6.78) | <0.0001 |
| Hyaline arteriolosclerosis | 2.36 (1.60-3.46) | <0.0001 |
| Cerebral amyloid angiopathy | 1.23 (0.64-2.37) | 0.53 |
| Siderocalcinosis | 1.51 (1.07-2.14) | 0.02 |
| Argyrophilic grain disease | 0.69 (0.47-1.01 | 0.06 |
| Lewy body disease (Braak Stage)  *. 0*  *. I-III*  *. IV-VI* | 1.0 (reference)  0.87 (0.45-1.66)  3.07 (1.74-5.41) | -  0.66  <0.0001 |

OR: odds ratio; CERAD: Consortium to Establish a Registry for Alzheimer's Disease; NFT: Neurofibrillary Tangle; NP: Neuropathological

Multivariable ordinal logistic regression model, adjusted for age, sex, education, and other neuropathological lesions in the table; dependent variable was three categories of Clinical Dementia Rating (CDR) score: CDR=0 (reference), CDR=0.5, and CDR ≥1
