# Supplementary material for: Neuropathological diagnoses and clinical correlates in older adults in Brazil: A cross-sectional study
Source: PLoS Med. 2017 Mar 28;14(3):e1002267. doi: 10.1371/journal.pmed.1002267 (PMC5369698; doi:10.1371/journal.pmed.1002267)
Supplement: S4 Table — (DOCX) [file pmed.1002267.s006.docx]

S4 Table. Association between neuropathological comorbidity score and cognitive outcomes (n=1,092)

|  | CDR-SB |  | IQCODE |  | NPI |  |
| --- | --- | --- | --- | --- | --- | --- |
|  | **Coefficient (95% CI)** | **p** | **Coefficient (95% CI)** | **p** | **Coefficient (95% CI)** | **p** |
|  |  |  |  |  |  |  |
| NPCS with Braak NFT score | 1.15 (1.04; 1.27) | <0.0001 | 0.12 (0.11; 0.14) | <0.0001 | 1.50 (1.14; 1.85) | <0.0001 |
|  |  |  |  |  |  |  |
| NPCS without AD pathology | 1.14 (0.86; 1.43) | <0.0001 | 0.11 (0.08; 0.14) | <0.0001 | 1.44 (0.54; 2.34) | 0.002 |
| Braak NFT Score  *Point assigned to Braak NFT stage III-IV*  *Point assigned to Braak NFT stage V-VI* | 1.19 (0.23; 2.14)  9.08 (7.67; 10.51) | 0.01  <0.0001 | 0.13 (0.03; 0.23)  1.01 (0.87; 1.16) | 0.01  <0.0001 | 3.00 (0.004; 5.99)  14.92 (10.42; 19.42) | 0.05  <0.0001 |
| Interaction term  *NPCS without AD pathology*Braak NFT III-IV*  *NPCS without AD pathology*Braak NFT V-VI* | 0.09 (-0.41; 0.60)  -0.40 (-0.95; 0.15) | 0.72  0.15 | 0.02 (-0.03; 0.07)  -0.04 (-0.10; 0.02) | 0.53  0.17 | -0.18 (-1.77; 1.42)  -1.48 (-3.20; 0.24) | 0.83  0.09 |

CDR-SB: Clinical Dementia Rating Sum of Boxes; IQCODE: Informant Questionnaire on Cognitive Decline in the Elderly; NPI: Neuropsychiatric Inventory; NPCS: Neuropathological Comorbidity Score; NFT: Neurofibrillary Tangle

Linear regression model adjusted for age, sex, and education
